# Cell-type specificity of Tim-3 in respiratory diseases: from mechanisms to clinical translation

**DOI:** 10.3389/fimmu.2026.1770761

**Published:** 2026-03-05

**Authors:** Qi Cui, Jinying Dou, Fukun Wang, Keran Jia

**Affiliations:** Clinical Laboratory, Bethune International Peace Hospital, Shijiazhuang, Hebei, China

**Keywords:** immune checkpoint, immune regulation, respiratory diseases, T cell exhaustion, TIM-3

## Abstract

As an immune checkpoint molecule, Tim-3 is expressed on T cells and other immune cells, and is crucial for maintaining immune homeostasis and preventing excessive inflammation. This article reviews the changes in expression, mechanism of action, and clinical significance of Tim-3 in major respiratory diseases such as asthma, chronic obstructive pulmonary disease (COPD), acute respiratory distress syndrome (ARDS), pulmonary infections, lung cancer, and pulmonary fibrosis. It emphasizes the dual role (protective and pathogenic) of Tim-3 in respiratory diseases and prospects its potential as a disease biomarker and a new target for immunotherapy.

## 1 Introduction

**Figure 1:**
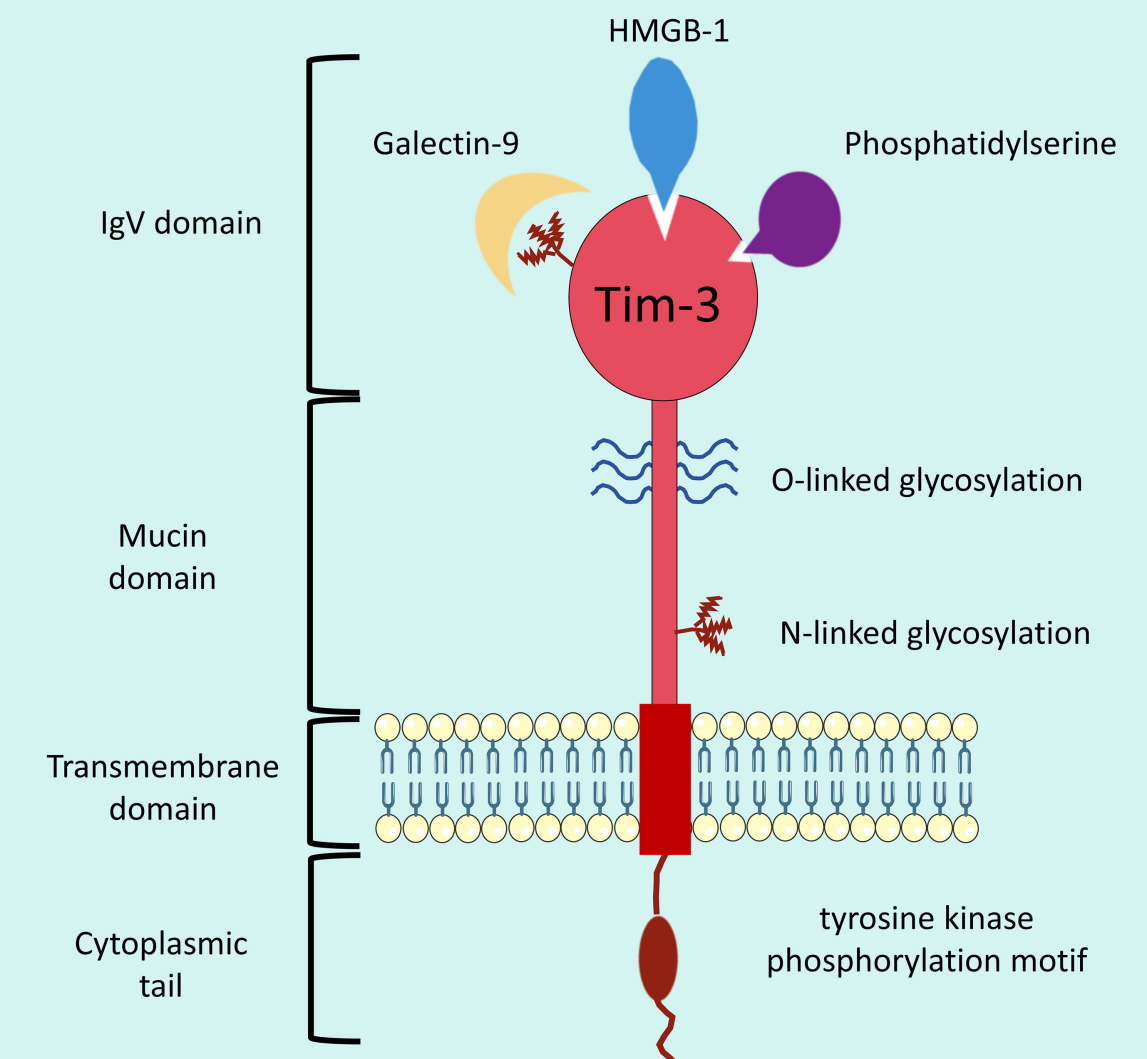
The structure of Tim-3 protein. Schematic diagram illustrating the structural composition and key functional components of the Tim-3 protein. Tim-3 is a transmembrane protein organized into four core domains (from extracellular to intracellular): The extracellular region comprises an IgV domain (the primary ligand-binding region, which interacts with ligands including Galectin-9, HMGB-1, and Phosphatidylserine) and a Mucin domain; Post-translational modifications (O-linked glycosylation in the IgV domain and N-linked glycosylation in the stalk region) are present in the extracellular segment; A Transmembrane domain anchors the protein to the cell membrane; The intracellular segment consists of a Cytoplasmic tail that contains a tyrosine kinase phosphorylation motif, which mediates downstream signal transduction.

T cell immunoglobulin and mucin domain-containing protein 3 (Tim-3) is a key checkpoint molecule in the immune regulatory network. Its encoding gene HAVCR2 is located on human chromosome 5 (5q33.1), and its protein structure includes an extracellular region (comprising an IgV-like domain and a mucin-like domain), a transmembrane region, and a cytoplasmic tail region. Among these, the IgV domain is the core functional region for binding to ligands, while the mucin domain is involved in protein glycosylation and intercellular adhesion (1, 2). Tim-3 exhibits cell-specific expression, mainly distributed on activated Th1 cells, cytotoxic T cells (CTLs), and regulatory T cells (Tregs). It is also expressed on the surface of innate immune cells such as macrophages and dendritic cells (DCs). Its expression level dynamically changes with the intensity of the immune response, serving as an important marker of immune cell activation and functional status (3) Currently identified Tim-3 ligands include galectin-9 (Gal-9), carcinoembryonic antigen-related cell adhesion molecule 1 (CEACAM1), high-mobility group box 1 (HMGB1), and phosphatidylserine (PtdSer). Binding to different ligands mediates different functions: Gal-9 binding mainly triggers immunosuppressive signals, CEACAM1 binding is involved in cytotoxicity regulation, and HMGB1 binding is associated with the regulation of inflammatory responses (4, 5) (**Figure 1**).

As a negative immune regulatory molecule, the core function of Tim-3 is to maintain immune homeostasis (6). In adaptive immunity, it prevents immunopathological damage by inhibiting overactivated Th1/CTL responses (7); in innate immunity, it regulates macrophage polarization and DC antigen-presenting function to balance pro-inflammatory and anti-inflammatory responses (8). The respiratory system, as an immune-active organ in direct contact with external pathogens and stimulants, has diseases (such as asthma, COPD, ARDS) whose pathogenesis mainly involves immune imbalance, such as Th1/Th2 imbalance, T cell exhaustion, and uncontrolled inflammation. This provides a pathological basis for Tim-3 to participate in disease regulation (9–11). The clinical success of targeting the PD-1/PD-L1 axis in oncology has validated immune checkpoint modulation as a powerful therapeutic strategy. Analogously, Tim-3 represents a next-generation checkpoint with broad relevance across both neoplastic and non-neoplastic respiratory diseases, where its cell-specific and stage-specific functions offer unique opportunities for intervention beyond the scope of PD-1/PD-L1 inhibition. While multiple immune checkpoints contribute to this complexity, Tim-3 warrants specific focus due to its direct, mechanistic involvement across diseases. For instance, the Tim-3/Gal-9 axis directly promotes Th2 bias in asthma (12, 13); in COPD, Tim-3 critically regulates CD8+ T-cell subset balance, and its deletion exacerbates disease in mice (14); in ARDS, Tim-3+ Tregs alleviate injury via the STAT3-IL-10 axis (15); and in lung cancer, Tim-3 drives the immunosuppressive niche in precancerous lesions (16). This article aims to systematically review the expression, mechanisms, and clinical significance of Tim-3 in major respiratory diseases, focusing on its cell-type and stage-specific regulatory roles, to provide a theoretical foundation for understanding disease immunopathology and developing Tim-3-targeted strategies (**Table 1**).

**Table 1:** Disease-specific summary of tim-3 roles.

Disease	Key tim-3 expression context	Primary functional role/mechanism	Clinical translation potential	Ref.
Allergic Asthma	↑ on peripheral blood CD4^+^ T cells; ↑ on epithelial mast cells in CRSwNP+asthma.	Promotes Th2 bias by inhibiting Th1 responses via Gal-9; role in mast cells under investigation.	Phenotype stratification biomarker (e.g., CRSwNP severity). Therapeutic targeting remains speculative.	(12, 13, 19, 20)
COPD	↑ plasma/soluble Tim-3 in stable phase; ↓ during exacerbation. Co-expressed with PD-1/TIGIT on CD8^+^ TEM.	Regulates macrophage M2 polarization; fine-tunes CD8^+^ Tc1/Tc17 balance; associated with T cell exhaustion.	Biomarker for differentiating stable vs. exacerbation phase. Potential target for modulating inflammation/exhaustion (preclinical).	(14, 28–30)
ALI/ARDS	↑ on Tregs (correlates with survival); etiology- and time-dependent expression on innate cells.	Protective: Treg STAT3-IL-10 axis. Damaging: CEACAM1-mediated NET formation in sepsis.	Prognostic biomarker (Treg Tim-3). Therapeutic target (e.g., Tim-3^+^ Treg transfer, Xuebijing), but etiology-critical.	(15, 36, 37, 39)
Pulmonary Fibrosis	Macrophages: Pro-fibrotic secretion vs. pro-resolution phagocytosis. Tregs: Anti-fibrotic.	Macrophage: Dual role (TGF-β1 secretion vs. apoptotic cell clearance via PtdSer). Treg: Mediates anti-fibrotic effect via IL-10.	Major challenge due to cell-type-opposing functions. Potential for Treg-specific Tim-3 enhancement.	(15, 44, 45)
Tuberculosis	↑ on T cells in MDR-TB & HIV-TB coinfection; marks exhausted and functional effector subsets.	"Threshold effect": Moderate expression supports anti-mycobacterial function; high/co-expression drives exhaustion.	Biomarker for MDR-TB and treatment response. Therapeutic target to reverse exhaustion (combined blockade).	(48, 50–52)
Lung Cancer	Early: Enriched in immune cells at lesion edge. Advanced: Co-expressed with PD-1 on exhausted CD8^+^ TILs.	Early: Establishes immunosuppressive niche via CEACAM1. Advanced: Mediates T cell exhaustion & ICI resistance.	Predictive biomarker for anti-PD-1 resistance. Target for precancerous interception & combo therapy in advanced disease.	(16, 61, 62, 67)

Abbreviations: ALI/ARDS, acute lung injury/acute respiratory distress syndrome; CRSwNP, chronic rhinosinusitis with nasal polyps; COPD, chronic obstructive pulmonary disease; ICI, immune checkpoint inhibitor; MDR-TB, multidrug-resistant tuberculosis; NET, neutrophil extracellular trap; TEM, terminally differentiated effector memory T cell; TIL, tumor-infiltrating lymphocyte; Treg, regulatory T cell.Symbol definitions: ↑ indicates an increase (upregulation or higher expression) in Tim-3 levels; ↓ indicates a decrease (downregulation or lower expression) in Tim-3 levels.

## 2 The role of Tim-3 in allergic asthma

### 2.1 Asthma pathology and immune landscape

Allergic asthma is a chronic airway disease driven by a dominant type 2 immune response. This is characterized by allergen-specific IgE production, eosinophilic inflammation, and a pronounced Th1/Th2 imbalance, with Th2 cells and their cytokines (e.g., IL-4, IL-5, IL-13) playing central roles in driving pathology (17, 18).

### 2.2 Expression characteristics and clinical correlations

Studies on patients with allergic asthma indicate that Tim-3 expression on immune cells is modulated in the context of allergic inflammation. The expression level of Tim-3 on peripheral blood CD4^+^T cells is significantly higher in patients than in healthy controls. This is associated with an increased co-expression frequency with PD-1 and correlates with the degree of Th1/Th2 imbalance, suggesting a link between allergen-driven immune activation and Tim-3 upregulation (12, 19).

Altered Tim-3 expression is also observed in specific clinical presentations. For instance, in asthma-like immune-related adverse events induced by immune checkpoint inhibitors, increased Tim-3 expression accompanies a rise in Th17 cells (20). Furthermore, in patients with chronic rhinosinusitis with nasal polyps (CRSwNP) complicated by asthma, the proportion of Tim-3^+^ mast cells in the epithelium positively correlates with postoperative disease severity scores, and this predictive value is unaffected by glucocorticoid treatment (20).

From a genetic perspective, single nucleotide polymorphisms (SNPs) of the HAVCR2 gene are associated with asthma susceptibility. Specific polymorphisms (e.g., +4259T>G, -574G>T, rs10515746) and genotypes have been linked to increased asthma risk in various populations (21–23), highlighting a genetic predisposition influencing Tim-3-related pathways.

The functional implications of these expression patterns are two-fold. First, the co-expression of Tim-3 with PD-1 on CD4^+^ T cells is associated with a dysregulated functional state that contributes to the pathogenic Th1/Th2 imbalance in asthma (12, 19). This state, while sharing features with exhaustion (e.g., impaired cytokine secretion), is primarily characterized within the allergic context by skewed differentiation and reinforced Th2 bias, rather than the profound loss of effector function typical of exhaustion in chronic infections or cancer. Second, the presence of Tim-3^+^ mast cells in the airway epithelium, which correlates with postoperative disease severity in patients with CRSwNP and asthma (20), directly links local Tim-3 expression to clinical disease burden, though the precise mechanistic contribution of Tim-3 signaling in mast cells to asthma pathology remains an area for further investigation.

### 2.3 Mechanism of action

Within the established Th2-dominant milieu of asthma, the upregulated Tim-3 contributes to pathogenesis by reinforcing the immune imbalance. Its primary mechanism involves the inhibition of Th1 cell activation and cytokine secretion (e.g., IFN-γ) via binding to its ligand Gal-9, thereby exacerbating the Th2 polarization central to allergic inflammation (13, 24). The expression frequency of Tim-3 on CD4^+^T cells shows a significant negative correlation with Th1/Th2 ratios, supporting this regulatory role (12). Additionally, Tim-3 signaling can impair the helper function of T cells for B cells; blocking Tim-3 *in vitro* reduces T cell-assisted production of IgG and IgE, likely by diminishing the secretion of Th2 cytokines like IL-4 (12).

While these *in vitro* findings suggest a role for Tim-3 in regulating T-cell-dependent B-cell responses, their direct relevance to the complex *in vivo* pathology of allergic asthma, involving multiple cell types and tissue compartments, requires further validation.

### 2.4 Research controversies and challenges

The pathogenic role of Tim-3 in asthma is context-dependent. A study using HAVCR2 gene-knockout mice found that Tim-3 deletion did not affect key features of ovalbumin-induced allergic airway inflammation, including eosinophilia, Th2 response, or IgE production (25). This indicates that Tim-3 may not be indispensable in all experimental models, highlighting complexities related to model systems, antigen types, or genetic backgrounds that must be considered for clinical translation.

The apparent non-essential role of Tim-3 in this specific mouse model presents a significant discrepancy with human correlative studies. Several hypotheses could explain this finding. First, model-specific factors may be at play; the OVA-induced murine model may not fully recapitulate the chronicity, complexity, or specific antigenic triggers of human allergic asthma, where Tim-3’s regulatory role might be more pronounced. Second, compensatory mechanisms within the intricate immune checkpoint network could mask the effect of Tim-3 deletion. Other inhibitory receptors (e.g., PD-1, LAG-3, TIGIT) might be upregulated or become more active in Tim-3 knockout mice, maintaining the overall threshold for immune regulation and disease manifestation. Third, the role of Tim-3 might be stage- or context-dependent, being more critical in the initial phases of sensitization, specific allergen challenges, or in the regulation of particular T cell subsets not fully assessed in the knockout model. These possibilities highlight that the pathogenic contribution of Tim-3 is likely not absolute but conditional, influenced by genetic background, environmental triggers, and the concurrent state of the immune system. Therefore, rather than negating its relevance, these contradictory findings underscore the complexity of Tim-3 biology and emphasize the need for careful contextual interpretation when translating findings from specific models to human disease.

### 2.5 Clinical implications

The expression level and genetic variants of Tim-3 hold promise as biomarkers for stratifying asthma phenotypes and assessing disease severity, particularly in complex cases like CRSwNP with asthma. However, its therapeutic targeting remains highly challenging and speculative due to its complex, context-dependent role in immune regulation, as evidenced by contradictory animal model data. Current clinical implications are primarily diagnostic rather than therapeutic.

## 3 The role of Tim-3 in chronic obstructive pulmonary disease

### 3.1 COPD pathology and immune landscape

COPD is characterized by persistent inflammation and tissue destruction in response to inhaled irritants like cigarette smoke. The immune pathology involves chronic activation of innate immune cells (macrophages, neutrophils) and adaptive responses, including increased lung infiltration of CD8+ T cells and Th1/Th17 cells, which contribute to alveolar damage and small airway fibrosis (26, 27).

### 3.2 Expression characteristics and association with disease stages

Chronic cigarette smoke exposure, the primary etiological factor for COPD, drives significant alterations in Tim-3 expression across the immune landscape. In patients, plasma Tim-3 levels are elevated in stable COPD but decrease during acute exacerbations (28). At the cellular level, smoke exposure in mice and humans upregulates Tim-3 on pro-inflammatory CD8+ T cell subsets (Tc1, Tc17) in the lungs (14), and leads to frequent co-expression of Tim-3 with PD-1 or TIGIT on peripheral blood CD8+ effector memory T cells (TEM) (29). Concurrently, smoke exposure reduces the proportion of pulmonary Tregs and downregulates the expression of the Tim-3 ligand Gal-9 on these cells (30).

These exposure-induced alterations in Tim-3 and its pathway are associated with clinical disease stages and functional immune impairment. The drop in plasma Tim-3 during exacerbations coincides with a broader pattern of dysregulated immune checkpoint signaling (28). The co-expression of Tim-3 with other inhibitory receptors on CD8+ TEM correlates with their weakened effector function (e.g., reduced IFN-γ secretion) (29). Furthermore, the smoke-induced loss of Gal-9 on Tregs is negatively correlated with lung function decline in patients (30), linking the altered Tim-3 pathway directly to disease severity.

### 3.3 Mechanism of action

The regulatory role of Tim-3 in COPD spans both innate and adaptive immunity, with distinct functions in specific immune cell subsets.

#### 3.3.1 Regulation of innate immunity and macrophage polarization

At the innate immunity level, Tim-3 is a key molecule promoting the polarization of macrophages to the anti-inflammatory M2 phenotype. *In vitro* studies indicate that treatment of macrophages with 1,25-dihydroxyvitamin D_3_ (1,25(OH)_2_D_3_) can significantly upregulate the expression of Tim-3. This upregulation is closely associated with the enhancement of the M2 phenotype, which promotes the secretion of anti-inflammatory cytokines IL-10 and TGF-β, while inhibiting the release of pro-inflammatory factors TNF and IL-6. This suggests that 1,25(OH)_2_D_3_ is expected to promote macrophage reprogramming to restore immune balance by regulating Tim-3, making it a potential candidate for COPD intervention (31).

#### 3.3.2 Regulation of CD4^+^ T cell responses

In adaptive immunity, Tim-3 plays a critical role in regulating CD4^+^ T cell function. Evidence from a COPD mouse model indicates that knockout of Tim-3 leads to further exacerbation of IFN-γ^+^ Th1 cell infiltration in the lungs and aggravated emphysema, identifying Tim-3 as an important negative feedback mechanism for limiting Th1 overactivation in this experimental system (14). Concurrently, the same model suggests that Tim-3 participates in pathogenesis by regulating the Th17/Treg balance, where Gal-9 derived from Tregs binds to Tim-3 on effector CD4^+^ T cells to inhibit Th17 differentiation (30). Translating to the human context, clinical data show that the smoke-induced decrease in Gal-9 expression on Tregs in patients is negatively correlated with enhanced Th17 responses and the decline in lung function (FEV1% predicted), supporting the pathological relevance of this axis (30).

#### 3.3.3 Regulation of CD8^+^ T cell subsets and exhaustion

In CD8^+^ T cells—key mediators of inflammation and tissue destruction—Tim-3 exerts complex regulation. Findings from mouse models show that Tim-3 deletion leads to a further increase in pro-inflammatory Tc1 cells while inhibiting Tc17 cells, disrupting subset homeostasis and promoting a shift toward terminal effector memory phenotypes (TEM) (14). In contrast, human studies reveal that the co-expression of Tim-3 with other inhibitory receptors (e.g., PD-1) on CD8^+^ effector memory T cells from COPD patients is associated with their functional exhaustion and reduced cytokine secretion (29). Thus, while murine data highlight Tim-3’s role in subset balance, human data strongly associate its expression with an exhausted T cell phenotype.

#### 3.3.4 Upstream regulators and microenvironmental crosstalk

Recent studies have identified key upstream regulators and microenvironmental influences on the Tim-3 pathway. Work in murine systems identified the transcription factor NFIL3 as a key upstream regulator of Tim-3 in CD4^+^ T cells, with knockout of Nfil3 downregulating Tim-3 and exacerbating inflammation (32). Furthermore, *in vitro* co-culture studies using human cells indicate that lung stromal cells from COPD patients can modulate the expression of molecules related to the Tim-3 signaling pathway in immune cells, suggesting a role for the tissue microenvironment in its regulation (29).

A study analyzing human plasma found that during acute exacerbations, the decrease in Tim-3 levels occurs alongside a broader pattern of dysregulated immune checkpoint molecules (e.g., lower PD-L2, higher CD86 and GITRL), suggesting Tim-3 is part of a dysregulated network during inflammatory outbreaks (28).

While mouse models have been instrumental in establishing causal relationships and elucidating mechanisms of Tim-3 in specific immune pathways, potential species-specific differences in Tim-3 ligand affinity, signaling cascades, and the nuanced development of T cell exhaustion necessitate cautious interpretation when translating these findings directly to human COPD pathophysiology.

### 3.4 Clinical implications

Dynamic changes in plasma Tim-3 levels may serve as a useful biomarker to differentiate between stable COPD and acute exacerbations. Therapeutically, targeting the Tim-3 pathway (e.g., via vitamin D analogues to promote M2 polarization) represents a novel conceptual strategy for modulating chronic inflammation and immune exhaustion. However, this approach remains in the preclinical exploration phase, and its net effect in patients requires careful evaluation due to the stage-specific roles of Tim-3.

### 3.5 Research perspectives

Future research should aim to disentangle the complex, stage-specific roles of Tim-3 in COPD pathogenesis. Key priorities include: 1) Mechanistic Deepening: Elucidating the precise signaling cascades downstream of Tim-3 in specific immune cell subsets (e.g., CD8^+^ Tc1 vs. Tc17 cells, M2 macrophages) and clarifying the regulatory details of axes like NFIL3/Tim-3. 2) Microenvironmental Crosstalk: Investigating how lung stromal cells and the inflammatory milieu influence Tim-3 expression and function via paracrine signals. 3) Translational Bridging: Validating plasma soluble Tim-3 (sTim-3) or cellular Tim-3 profiles as robust biomarkers for distinguishing disease stages (stable vs. exacerbation) and predicting progression, through well-designed longitudinal clinical studies. 4) Therapeutic Modeling: Preclinical studies should test the net effect of Tim-3 modulation (agonism vs. antagonism) at different disease stages and in combination with other pathways (e.g., PD-1, cytokine inhibition) to identify safe and effective therapeutic windows. 5) Comparative Checkpoint Biology: Investigate the comparative and potentially synergistic roles of Tim-3 and PD-1 pathways in COPD. While PD-1 is also associated with T-cell exhaustion in COPD, understanding whether Tim-3 operates in parallel, sequentially, or in distinct cell subsets could inform rational combination therapies, analogous to strategies being pursued in oncology.

## 4 The role of Tim-3 in acute lung injury/acute respiratory distress syndrome

### 4.1 ALI/ARDS pathology and immune landscape

Acute lung injury (ALI) and its severe form, acute respiratory distress syndrome (ARDS), are life-threatening conditions characterized by alveolar-capillary barrier disruption, diffuse pulmonary inflammation, and refractory hypoxemia, with mortality rates reaching 30%–50% (33–35). Immune dysregulation is the core pathogenic mechanism, featuring a dysregulated, excessive innate immune response dominated by rapid neutrophil infiltration and a ‘cytokine storm’, which leads to diffuse alveolar damage, endothelial/epithelial injury, and pulmonary edema.

### 4.2 Expression characteristics and association with prognosis

From the perspective of expression characteristics and prognostic association, a study on ALI patients found that the expression level of Tim-3 on peripheral blood CD4^+^CD25^+^FoxP3^+^Tregs is significantly higher than that in healthy controls, and the levels of Tregs and Tim-3 expression in surviving patients at admission are significantly higher than those in deceased patients, suggesting that Tim-3 may affect patient prognosis by enhancing the immunosuppressive function of Tregs (36). Animal experiments further verified this association: in malaria- or LPS-induced ALI models, the number of Tim-3^+^cells in lung tissue and mediastinal lymph nodes increases significantly with disease progression, and is positively correlated with the degree of lung tissue inflammation, indicating that Tim-3 expression can be used as a potential biomarker for evaluating the severity and prognosis of ALI/ARDS (15, 37). Notably, the functional consequence of this increased expression is not static but exhibits critical time-dependence, with the Tim-3/Galectin-9 axis acting as a dynamic switch that can promote resolution in early inflammation but contribute to dysregulation if sustained, as detailed in the following section.

### 4.3 Mechanism of action and etiology dependence

The function of Tim-3 in ALI/ARDS is not universally protective or damaging but is instead determined by a critical triad of factors: the cell type expressing Tim-3 (e.g., Tregs vs. neutrophils), the underlying disease etiology (e.g., sepsis vs. malaria vs. direct lung injury), and the temporal stage of inflammation. The following mechanisms illustrate this profound context-dependence, explaining how Tim-3 can contribute to resolution or exacerbation of injury depending on the specific pathological context. In Treg-mediated immune regulation, Tim-3^+^Tregs can promote the secretion of IL-10 by activating the STAT3 signaling pathway, thereby inducing the polarization of macrophages to the anti-inflammatory M2 phenotype and reducing the expression of pro-inflammatory M1 markers. Neutralization of IL-10 or inhibition of STAT3 can reverse this effect, indicating that Tim-3^+^Tregs play a key role in the regulation of ALI inflammation through the STAT3-IL-10 axis (15). At the same time, the Tim-3/Gal-9 pathway shows a time-dependent regulatory characteristic: short-term LPS stimulation can enhance the binding of the two to inhibit M1 polarization, while long-term stimulation leads to the inactivation of the pathway and uncontrolled inflammation. This time dependence provides a mechanistic reference for understanding the inflammatory changes in different stages of ALI (38).

In addition, Tim-3 can synergize with CEACAM1 to promote the formation of neutrophil extracellular traps (NETs), aggravating lung tissue damage. However, the traditional Chinese medicine Xuebijing can inhibit NETs by downregulating the expression of the two, thereby alleviating sepsis-induced ALI, suggesting that the Tim-3/CEACAM1-NETs axis may become a potential target for ALI treatment (39); in the regulation of Th cell balance, inhibition of the Tim-3/Gal-9 axis can correct the Th1/Th2/Th17 imbalance in ALI and reduce the secretion of pro-inflammatory factors, further confirming the regulatory role of Tim-3 in ALI immune disorders (40).

It is worth noting that the role of Tim-3 in ALI/ARDS of different etiologies shows significant differences, presenting an etiology-dependent “protective-damaging” characteristic. In sepsis-induced ALI, Tim-3 promotes the formation of NETs by binding to CEACAM1, mainly exerting a pro-damaging effect (39); in malaria infection-induced ALI, the Tim-3/Gal-9 pathway can inhibit the excessive secretion of type I interferon by macrophages, limiting the spread of inflammation and showing a protective effect, and blocking this pathway will aggravate lung tissue damage (37, 41); in LPS- or trauma-induced ALI, the immune regulatory effect of Tim-3^+^Tregs is dominant, which alleviates lung damage by promoting neutrophil clearance and inhibiting fibrosis (15, 38).

### 4.4 Targeted strategies and potential

Based on the regulatory role of Tim-3, a variety of targeted strategies have shown therapeutic potential in animal experiments. Adoptive transfer of Tim-3^+^Tregs can significantly improve the survival rate of ALI mice, reduce lung inflammation and fibrosis, and has a wider therapeutic window than unsorted Tregs, suggesting that Tim-3^+^Tregs may become a preferred option for ALI cell therapy (15); combined use of NAC and dexmedetomidine can downregulate the expression of Tim-3/Gal-9 and synergistically correct Th cell imbalance, providing ideas for combined drug therapy of ALI (40); the traditional Chinese medicine Xuebijing inhibits NETs by targeting the Tim-3/CEACAM1 axis, improving lung function in sepsis-induced ALI, demonstrating the potential of traditional Chinese medicine in Tim-3-targeted therapy (39). Current studies still need large-sample clinical verification of the biomarker value of Tim-3 and clarification of precise intervention strategies under different etiologies to promote its clinical translation.

### 4.5 Clinical implications

Tim-3 expression on immune cells, particularly Tregs, shows prognostic value in ARDS, with higher levels correlating with better survival. This positions Tim-3 as a potential prognostic biomarker. Therapeutically, strategies like adoptive transfer of Tim-3+ Tregs or pharmacologic modulation of the Tim-3/Gal-9 axis (e.g., with Xuebijing) have shown promise in preclinical models (39). A critical challenge for clinical translation is the etiology-dependent duality of Tim-3’s role, demanding precise patient stratification.

## 5 The role of Tim-3 in pulmonary fibrosis

### 5.1 Pulmonary fibrosis pathology and immune landscape

Pulmonary fibrosis results from aberrant wound healing following lung injury, leading to excessive scar formation. The immune dysregulation is skewed towards a pro-fibrotic state, characterized by the polarization of macrophages to an M2 phenotype and the dominant action of mediators like TGF-β, which drive fibroblast activation and collagen deposition (42, 43).

### 5.2 Expression characteristics and cell specificity

The role of Tim-3 in pulmonary fibrosis shows significant cell specificity, and its function depends on the type of expressing cells and the disease microenvironment. This specificity is the core for understanding the complex role of Tim-3 in pulmonary fibrosis. In alveolar macrophages, the expression status of Tim-3 directly affects the process of pulmonary fibrosis. Wang et al. found in a bleomycin (BLM)-induced mouse model of pulmonary fibrosis that overexpression of Tim-3 in macrophages can promote the secretion of pro-fibrotic factors such as TGF-β1 and IL-10, aggravating lung tissue pathological damage and collagen deposition. Moreover, the pro-fibrotic effect mediated by Tim-3 disappears after macrophage depletion, which clearly indicates that Tim-3 derived from macrophages is an important trigger for pulmonary fibrosis (44).

### 5.3 Mechanism of action: cell type-dependent bidirectional regulation

However, Tim-3 on alveolar macrophages does not only play a pro-fibrotic role. Isshiki et al. further confirmed that Tim-3 on the surface of alveolar macrophages can mediate the phagocytosis of apoptotic cells by binding to phosphatidylserine. Treatment with anti-Tim-3 antibodies can inhibit this phagocytic process, leading to the accumulation of apoptotic cells, upregulation of TGF-β1, and downregulation of the anti-fibrotic factor HGF, which in turn exacerbates pulmonary fibrosis (45). This finding indicates that Tim-3 on alveolar macrophages has dual potential of “pro-fibrosis” and “anti-fibrosis protection”, and its core depends on whether Tim-3 is involved in the key process of apoptotic cell clearance. The microenvironment where cells are located (such as the number of apoptotic cells and the level of pro-inflammatory/anti-inflammatory factors) may be the key factor determining its functional direction.

**Figure 2:**
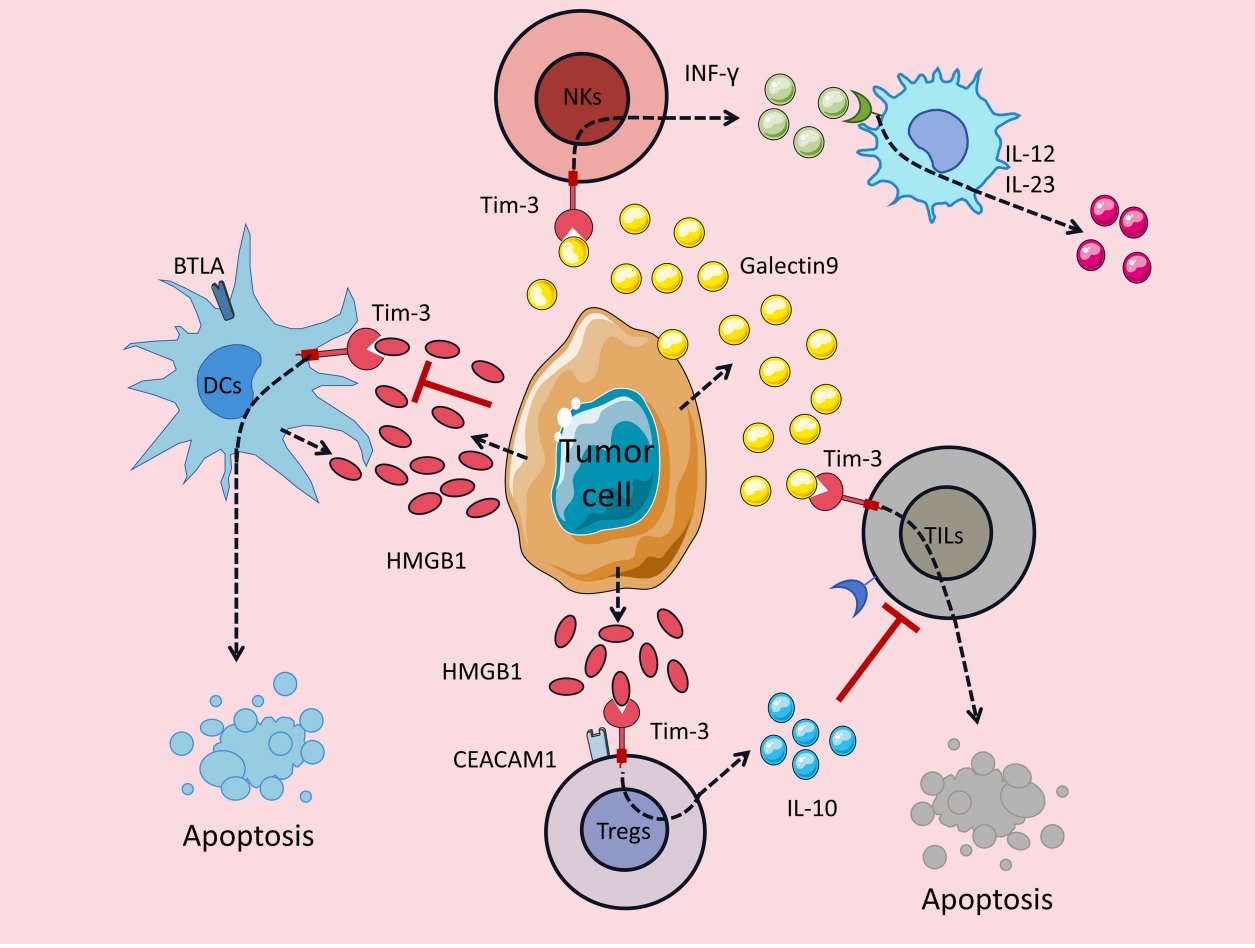
Cell-specific and bidirectional regulatory roles of Tim-3 in pulmonary fibrosis. Schematic diagram illustrating the dual regulatory roles of Tim-3 in pulmonary fibrosis. On one hand, Tim-3 expressed on alveolar macrophages mediates phagocytosis of apoptotic cells, thereby inhibiting pulmonary fibrosis; however, excessive Tim-3 on macrophages can promote the secretion of pro-fibrotic factors (e.g., TGF-β1, IL-10), leading to accumulation of macrophages, activation and proliferation of fibroblasts into myofibroblasts, and subsequent extracellular matrix (collagen) deposition that exacerbates fibrosis. On the other hand, Tim-3+ regulatory T cells (Tregs) activate the STAT3 signaling pathway to secrete IL-10, which induces polarization of M1 macrophages to anti-inflammatory M2 macrophages, inhibits the secretion of pro-inflammatory cytokines (e.g., TNF-α, IL-6), and ultimately suppresses pulmonary fibrosis. Monocyte/macrophage dysfunction and alveolar epithelial cell injury further contribute to the fibrotic process by promoting myofibroblast differentiation.

Different from alveolar macrophages, the expression of Tim-3 in regulatory T cells (Tregs) shows a clear anti-pulmonary fibrosis effect, which is in sharp contrast to its function in macrophages. Liu et al. found in a lipopolysaccharide (LPS)-induced pulmonary fibrosis model after acute lung injury (ALI) that Tim-3^+^Tregs activate the STAT3 pathway by secreting IL-10, induce the polarization of macrophages to the M2 phenotype, reduce the release of pro-inflammatory factors (IL-6, TNF-α), and ultimately reduce lung tissue collagen deposition and fibrosis degree (15); more importantly, Tim-3^-^Tregs lose this regulatory ability. This result clearly suggests that Tim-3 is a key molecule for Tregs to exert anti-fibrotic functions, and whether Tim-3 is expressed on Tregs directly determines the regulatory effect of this cell population on pulmonary fibrosis (15) (**Figure 2**).

### 5.4 Targeted strategies and principles

Comprehensive analysis of existing studies shows that the role of Tim-3 in pulmonary fibrosis has “cell type dependence” and “functional complexity”, and it cannot be simply defined as a pro-inflammatory or anti-inflammatory molecule. In clinical practice, when targeting Tim-3 for the treatment of pulmonary fibrosis, the type of cells expressing Tim-3 should be clarified first: the regulation of Tim-3 in macrophages needs to strictly distinguish between the pro-fibrotic factor secretion function and the apoptotic cell phagocytosis function to avoid blind blocking; on the contrary, enhancing the expression or activity of Tim-3 on Tregs may become a potential strategy for inhibiting pulmonary fibrosis, such as improving the expression level of Tim-3 in Tregs through drug regulation or cell engineering to enhance its anti-fibrotic effect. Future studies need to further analyze the molecular mechanism of Tim-3 exerting functions in different cell types, so as to provide a more sufficient theoretical basis for precise targeted therapy.

### 5.5 Clinical implications

The clinical implications of Tim-3 in pulmonary fibrosis are complex and not yet defined for direct application. Its value as a biomarker is uncertain due to its cell-specific dual functions. Therapeutically, targeting Tim-3 is highly challenging; a universal agonist or antagonist strategy is likely to fail. Future interventions would require exquisite cell-type selectivity—for example, enhancing Tim-3 function specifically on Tregs while sparing or modulating its role on macrophages—a significant hurdle for current drug development.

## 6 The role of Tim-3 in pulmonary tuberculosis

### 6.1 Tuberculosis pathology and immune landscape

The immunopathology of pulmonary tuberculosis (TB) centers on the granuloma, an organized structure formed to contain Mycobacterium tuberculosis. The role of Tim-3 has been most extensively studied in TB among pulmonary infections. This focus is largely due to TB’s chronic nature, which involves a delicate and protracted interplay between host protective immunity and bacterial evasion strategies—a process where checkpoint molecules like Tim-3 are critically engaged (46). In contrast, acute bacterial pneumonias (e.g., pneumococcal) are typically resolved rapidly by innate and acute adaptive responses, where the role of regulatory checkpoints like Tim-3 is less characterized and may be more transient. Protective immunity relies on robust Th1-cell responses, while disease progression is often associated with bacterial evasion strategies and eventual T-cell exhaustion. As a key immune checkpoint molecule, Tim-3 plays a complex and core regulatory role in this process. Its expression level, gene polymorphisms, and mediated signaling pathways profoundly affect the host’s anti-infective immune response, disease susceptibility, severity, and treatment outcome (47).

### 6.2 Expression characteristics and clinical correlations

In terms of expression characteristics and clinical correlations, Tim-3 expression in TB patients is closely linked to the immune functional state and treatment response. The expression levels of Tim-3 on peripheral blood CD4^+^ and CD8^+^ T cells are significantly higher in patients with multidrug-resistant tuberculosis (MDR-TB) than in those with drug-susceptible tuberculosis (NR-TB). This elevated expression is closely associated with an advanced T cell exhaustion phenotype—a state of immune dysfunction resulting from persistent antigen exposure in chronic, refractory infections like MDR-TB—manifested as decreased T-cell proliferative capacity and reduced effector cytokine secretion (48).

Genetic factors also affect the role of Tim-3 in pulmonary infections. Polymorphisms of the HAVCR2 gene (such as rs13170556) are associated with TB susceptibility in a gender-dependent manner. Specific genotypes in men can significantly increase the risk of onset, while no similar association is found in women. This reveals the complex mechanism by which genetic factors affect the host’s immune response to Mycobacterium tuberculosis (M. tb) by regulating Tim-3 expression (49). In the context of coinfection with immunodeficiency, the immunosuppression mediated by Tim-3 is particularly prominent. The gene and protein expression levels of Tim-3 in peripheral blood mononuclear cells (PBMCs) of patients with HIV-TB coinfection are significantly higher than those in patients with HIV alone. Moreover, the frequency of Tim-3^+^T cells is negatively correlated with the peripheral blood CD4^+^T cell count and positively correlated with the HIV viral load, indicating that Tim-3 may inhibit T cell function while promoting HIV replication and persistent M. tb infection, forming a “immunosuppression-dual pathogen coexistence” vicious cycle and aggravating the disease process (50).

### 6.3 Mechanism of action

In terms of mechanism, Tim-3 directly participates in the pathophysiological process of pulmonary infections by finely regulating the functions of different immune cell subsets. For T cell function, Tim-3 exhibits a “threshold effect”: on the one hand, Tim-3^+^CD4^+^and Tim-3^+^CD8^+^T cells in the peripheral blood of patients with active tuberculosis (ATB) show a stronger ability to inhibit the replication of M. tb in macrophages, and blocking the Tim-3/Gal-9 axis significantly reduces the secretion of IFN-γ and TNF-α by these T cells, suggesting that moderately expressed Tim-3 may play a supporting role in maintaining the anti-M. tb effector function of T cells (51); on the other hand, when Tim-3 is continuously highly expressed, especially when co-expressed with exhaustion markers such as PD-1 and CD39, T cell function is severely impaired. In ATB patients, the cytotoxic function of CD8^+^T cells is weakened due to the co-expression of Tim-3 and PD-1, and CD4^+^CXCR5^+^T cells (precursors of follicular helper T cells) also have impaired proliferation ability and IL-21 secretion due to the co-expression of the two, thereby affecting B cell helper function. Combined blocking of Tim-3 and PD-1 can significantly restore the function of such T cells, proving that Tim-3 is a key molecule mediating T cell exhaustion (52).

In the local pulmonary infection microenvironment, Tim-3 shows an “enhancing” characteristic on the function of certain effector cells. In tuberculous pleural effusion, the proportion of the Tim-3^+^CD69^+^CD103^+^subset of tissue-resident mucosal-associated invariant T (MAIT) cells is significantly increased. This subset has a stronger ability to secrete IFN-γ and granzyme B than Tim-3^-^MAIT cells, and its frequency is positively correlated with the scope of TB lesions, suggesting that Tim-3 may mark a group of effector cells with active functions and involved in local infection control (53); at the same time, cytokines IL-2, IL-12p70, and IL-18 can regulate the expression of Tim-3 on MAIT cells, and this subset has unique lipid metabolism characteristics, which may support its continuous function in the inflammatory environment (53).

M. tb itself can also actively use the Tim-3 pathway to escape immune clearance. Its secreted protein MPT64 can induce dendritic cells (DCs) to differentiate into myeloid-derived suppressor cells (MDSCs) with high expression of Tim-3 and PD-L1. These cells create an immunosuppressive microenvironment for M. tb by promoting the generation of regulatory T cells (Tregs) and inhibiting the differentiation of Th1/Th17 cells (54); at the same time, the cell wall component lipoarabinomannan (LAM) of M. tb can downregulate the Tim-3 ligand Gal-9 on the surface of macrophages, thereby weakening the anti-inflammatory effect mediated by the Tim-3/Gal-9 axis and the bactericidal function of macrophages, and reducing the expression of TNF and its receptor TNFR2, further inhibiting pathogen clearance ability (55). In non-tuberculous mycobacterial pulmonary disease (NTM-PD), the proportion of Tim-3^+^Tregs in local lung tissue increases, which inhibits the activation of effector T cells through the Tim-3/Gal-9 pathway, leading to prolonged inflammation and decreased lung function (56).

### 6.4 Targeted strategies and precautions

Given the core role of Tim-3 in pulmonary infections, targeting Tim-3 has become a potential immunotherapeutic strategy. Thymosin α1 combined with standard anti-TB regimens can significantly reduce the mRNA level of Tim-3 in the peripheral blood of patients and promote Th1-type cytokine responses, suggesting that immunomodulators may restore the anti-TB function of T cells by inhibiting the Tim-3 pathway (57). However, Tim-3-targeted strategies need to have high context specificity. In the T cell exhaustion stage caused by chronic infection (such as HIV-TB coinfection and MDR-TB), combined blocking of Tim-3 and checkpoints such as PD-1 may help reverse T cell exhaustion and restore pathogen clearance efficiency; in the acute infection stage or local microenvironment, for effector cells such as Tim-3^+^MAIT cells, intervention strategies need to be carefully evaluated to avoid weakening their protective immune function. In addition, the BCG vaccine, as a core means for TB prevention, has no significant regulatory effect on Tim-3 expression when stimulating monocytes of healthy adults or newborns, indicating that the protective immunity induced by the BCG vaccine may be relatively independent of the Tim-3 pathway. When formulating Tim-3-targeted strategies, the basic protective effect of the vaccine should not be ignored (58).

### 6.5 Clinical implications

In tuberculosis, Tim-3 expression is a compelling biomarker for identifying T-cell exhaustion states, differentiating MDR-TB, and monitoring treatment response. Therapeutically, blocking Tim-3 (especially in combination with anti-PD-1) is a promising strategy to reverse T-cell exhaustion in chronic infections like MDR-TB or HIV-TB co-infection. Crucially, this must be approached with caution, as timing is critical to avoid impairing protective immune responses during acute infection phases.

## 7 The role of Tim-3 in lung cancer

### 7.1 The immunosuppressive tumor microenvironment in lung cancer

The lung cancer microenvironment is profoundly immunosuppressive, facilitating tumor immune evasion through the functional exhaustion of tumor-infiltrating CD8+ T cells and the accumulation of regulatory cells like Tregs and tumor-associated macrophages (TAMs) (59). Within this context, Tim-3 exhibits obvious ‘stage-specific’ expression and function, ranging from driving precancerous lesions to mediating late treatment resistance, positioning it as an important target in lung cancer immunotherapy (60).

### 7.2 Expression characteristics and association with disease stages

In precancerous lesions and early-stage lung cancer, Tim-3 plays a role in driving lesion progression. Studies have confirmed that in lung adenocarcinoma precancerous lesions such as atypical adenomatous hyperplasia (AAH) and adenocarcinoma *in situ* (AIS), Tim-3 is not uniformly expressed but is significantly enriched in immune cells at the edge of the lesions, including CD4^+^ memory T cells, conventional dendritic cells (cDCs), macrophages, and myeloid-derived suppressor cells. Its expression level increases with the elevation of lesion malignancy, suggesting a close correlation with disease progression (16). What is particularly important is that carcinoembryonic antigen-related cell adhesion molecule 1 (CEACAM1), a ligand expressed by epithelial cells, interacts with Tim-3 on myeloid cells, forming an epithelial-myeloid cell communication bridge and promoting the formation of an early immunosuppressive microenvironment (16). Animal experiments further confirm that blocking Tim-3 in the precancerous stage can enhance the antigen-presenting ability of dendritic cells, promote the polarization of pro-inflammatory M1-type macrophages, and inhibit the polarization of pro-tumor M2-type macrophages, thereby significantly delaying the progression of lesions to invasive lung adenocarcinoma. However, blocking Tim-3 in established invasive cancer has limited efficacy, which highlights the unique value of Tim-3 as a potential target for “precancerous intervention” (16).

### 7.3 Mechanism of action

In advanced non-small cell lung cancer (NSCLC), the core role of Tim-3 shifts to mediating T cell function exhaustion and becomes a key driver of immune checkpoint inhibitor (ICI) resistance. Tim-3 is often co-expressed with inhibitory receptors such as PD-1, LAG-3, and TIGIT on the surface of tumor-infiltrating CD8^+^T cells. Among them, the Tim-3^+^PD-1^+^phenotype marks terminally exhausted T cells, whose ability to secrete effector cytokines such as IFN-γ and TNF-α is severely impaired. The frequency of such cells is significantly correlated with shortened progression-free survival of patients and reduced objective response rate of ICI treatment (61–63). In contrast, Tim-3-negative CD8^+^T cells (such as TCF1^+^PD-1^+^pre-exhausted subsets) retain partial proliferation and effector functions. In the T cell exhaustion signature gene set constructed by transcriptome analysis, the Tim-3-encoding gene HAVCR2 is a core component, further confirming its central position in the exhaustion program (63).

Tim-3 also regulates the lung cancer immunosuppressive microenvironment in multiple dimensions, becoming a key immunosuppressive “node”. At the dendritic cell level, Tim-3 inhibits the activation of the cGAS-STING pathway in DCs by binding to ligands Gal-9 and HMGB1, reducing the uptake of extracellular DNA, thereby weakening its antigen-presenting function and indirectly leading to insufficient activation of CD8^+^T cells (63); at the tumor-associated macrophage (TAM) level, highly expressed Tim-3 on the surface can promote the polarization of TAMs to the M2 phenotype through the STAT3-IL-10 axis, thereby secreting anti-inflammatory factors such as IL-10 and TGF-β and aggravating immunosuppression (64).

**Figure 3:**
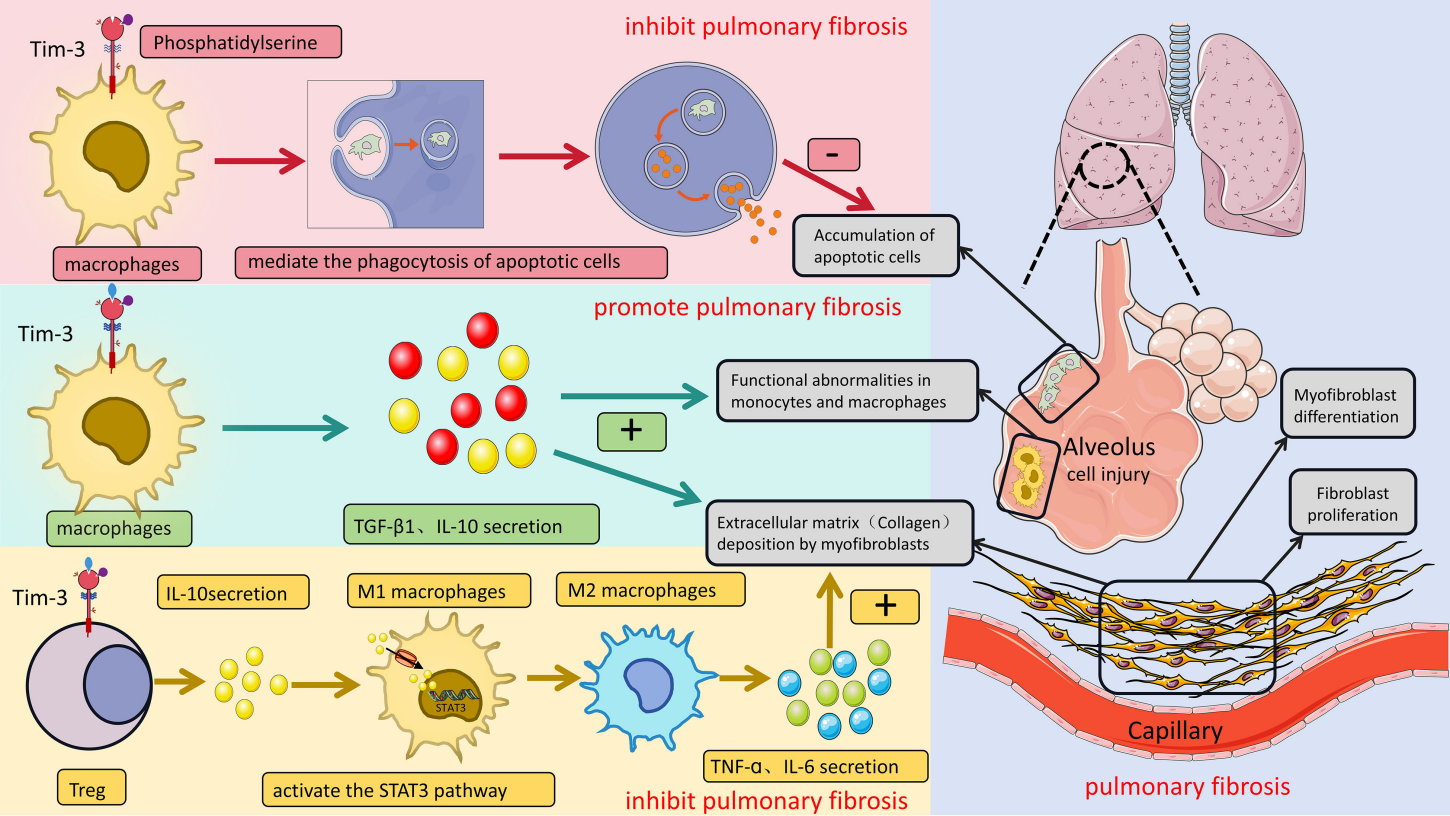
The multi-ligand network of Tim-3 in the lung cancer immunosuppressive microenvironment. Tim-3 integrates signals from multiple ligands to coordinate immunosuppression.① Binding of Galectin-9 (Gal-9) to Tim-3 on dendritic cells (DCs) inhibits their maturation and IL-12 production.② Gal-9 engagement on T cells induces apoptosis and exhaustion.③ High Mobility Group Box 1 (HMGB1) released from tumor cells ligates Tim-3, further suppressing T cell function.④ Interaction with Carcinoembryonic Antigen-Related Cell Adhesion Molecule 1 (CEACAM1) delivers a strong co-inhibitory signal.⑤ Tim-3^+^ regulatory T cells (Tregs) utilize this axis to enhance suppression via IL-10.⑥ The Tim-3/BTLA pathway may contribute to natural killer (NK) cell dysfunction. This network positions Tim-3 as a central regulator of immune evasion in lung cancer. Abbreviations: DC, dendritic cell; Treg, regulatory T cell; NK, natural killer cell; Gal-9, Galectin-9; HMGB1, High Mobility Group Box 1; CEACAM1, Carcinoembryonic Antigen-Related Cell Adhesion Molecule 1; BTLA, B and T lymphocyte attenuator.

External factors also affect the expression and function of Tim-3, such as the hypoxic environment. In patients with lung cancer complicated with obstructive sleep apnea (OSA), intermittent hypoxia significantly upregulates the expression of Gal-9 and Tim-3 on T cells by activating the HIF-1 pathway. The plasma level of soluble Tim-3 (sTim-3) is positively correlated with tumor invasiveness and patient mortality (65); in addition, tumor necrosis factor-α (TNF-α) can upregulate HMGB1, promote its binding to Tim-3, and activate the NF-κB pathway, exacerbating CD8^+^T cell exhaustion and leading to anti-PD-1 treatment resistance (66). This represents a fundamental shift from its role in precancerous lesions, where Tim-3 acts to establish an immunosuppressive niche, to its function in advanced disease, where it acts to maintain T-cell exhaustion and drive therapy resistance (**Figure 3**).

### 7.4 Targeted therapeutic strategies and challenges

In terms of targeted therapeutic strategies and challenges, targeting Tim-3 provides a new direction for lung cancer treatment, especially for patients with PD-(L)1 resistance. Clinical studies have shown that the combination of the anti-Tim-3 monoclonal antibody Cobolimab and the anti-PD-1 monoclonal antibody Dostarlimab has controllable safety in patients with advanced NSCLC who have progressed after PD-(L)1 treatment. Moreover, patients with high Tim-3 expression in pre-treatment tumor tissues have significantly better objective response rates and disease control rates, suggesting that Tim-3 can be used as a predictive biomarker for combination therapy (67). However, the monotherapy efficacy of Tim-3 is limited, which may be related to the compensatory activation of other pathways such as LAG-3 and TIGIT (62). Small-molecule Tim-3 inhibitors (such as HIT104310526) block the binding of Tim-3 to ligands through π-π stacking, which can restore CD8^+^T cell cytotoxicity and show selectivity for NSCLC cells, and overcome the tissue penetration limitation of monoclonal antibodies. However, water solubility and potential cardiotoxicity (such as hERG channel inhibition risk) still need to be optimized, and local administration (such as nebulization) may be used to increase tumor drug concentration in the future (64).

The development path for Tim-3 inhibitors invites comparison with that of PD-(L)1 inhibitors. While PD-(L)1 blockade primarily reverses T-cell exhaustion, Tim-3 modulation may offer additional layers of intervention, such as reprogramming the myeloid compartment and disrupting precancerous niches. However, several key challenges distinct from the PD-1 pathway must be overcome for successful Tim-3 inhibitor development. First, the biology is more complex: Tim-3 has multiple ligands (e.g., Gal-9, CEACAM1, HMGB1) mediating context-dependent signals, unlike the more focused PD-1/PD-L1/L2 axis. This necessitates a deeper understanding of the dominant ligand-repair in specific disease stages or patient subsets to predict agonist vs. antagonist effects. Second, predictive biomarkers are less defined. While PD-L1 expression (despite limitations) guides therapy, a robust biomarker for Tim-3 inhibitor response is still emerging, with pre-treatment Tim-3 expression on tumor-infiltrating immune cells being a candidate. Third, mechanisms of primary/adaptive resistance may differ; for instance, compensatory upregulation of LAG-3 or TIGIT upon Tim-3 blockade has been observed. Finally, drug design faces unique hurdles: small-molecule inhibitors must achieve high specificity to avoid off-target effects (e.g., hERG channel inhibition), and antibody-based therapies must consider potential Fc-mediated effector functions that could deplete Tim-3+ immune cells. Addressing these gaps—through continued mechanistic dissection, biomarker validation in clinical trials, and innovative drug engineering—is essential to translate the promise of Tim-3 targeting into clinical success akin to that of PD-(L)1 inhibitors.

### 7.5 Clinical implications

In lung cancer, Tim-3 is a valuable predictive biomarker, especially for resistance to PD-1/PD-L1 inhibitors. Its expression in precancerous lesions also highlights its potential as a target for interception therapy. Therapeutically, anti-Tim-3 monoclonal antibodies (e.g., Cobolimab) are now in clinical trials, primarily in combination with anti-PD-1 for advanced NSCLC. The key clinical challenge is overcoming primary and adaptive resistance to achieve efficacy beyond current checkpoint inhibitors.

## 8 Summary and outlook

### 8.1 Summary of core roles

A central theme emerging from this review is that the function of Tim-3 in respiratory diseases—whether protective or pathogenic—is not intrinsic but is defined by a triad of contextual determinants. First, the expressing cell type fundamentally dictates outcome; for instance, Tim-3 on Tregs often mediates anti-inflammatory resolution, whereas on alveolar macrophages or neutrophils its role can be pro-fibrotic or tissue-damaging. Second, the specific disease context (e.g., allergic asthma vs. chronic infection vs. cancer) engages Tim-3 in distinct pathological circuits, from reinforcing Th2 bias to driving T-cell exhaustion. Third, the disease stage and microenvironmental cues (e.g., acute vs. chronic phase, hypoxia, ligand availability) critically influence whether Tim-3 signaling thresholds promote homeostasis or exacerbate pathology. This context-dependency underpins the ‘double-edged sword’ nature of Tim-3 and necessitates a precise, stratified approach for any diagnostic or therapeutic application.

The role of Tim-3 in respiratory diseases shows significant “three specificities”. The first is disease specificity: in asthma, Tim-3 mainly drives Th2 bias by inhibiting Th1 cell function, participating in the occurrence and development of allergic inflammation; in COPD, Tim-3 acts as an immune regulatory hub, maintaining chronic inflammatory homeostasis by regulating macrophage polarization and T cell (CD4^+^, CD8^+^) function balance, and its expression changes dynamically with disease stages; in ARDS, the role of Tim-3 shows “protective-damaging” duality depending on the etiology, promoting damage in sepsis-induced cases and potentially exerting a protective effect in malaria- or LPS-induced cases; in pulmonary infections, Tim-3 is a key molecule for host anti-infective immunity and pathogen escape, with moderate expression supporting T cell effector functions and excessive expression leading to T cell exhaustion; in lung cancer, Tim-3 has both functions of driving precancerous lesions and mediating late treatment resistance; in pulmonary fibrosis, the function of Tim-3 varies with cell types, showing bidirectionality on alveolar macrophages and anti-fibrotic effect on Tregs (**Table 2**).

**Table 2:** Tim-3 function by immune cell type.

Immune cell type	General function of tim-3	Context-specific examples from respiratory diseases	Key ligands/pathways	Ref.
CD4^+^ T Cells (Effector)	Inhibitory regulation; modulates differentiation.	Asthma: Inhibits Th1, promoting Th2 bias. COPD: Limits excessive Th1 inflammation. TB: High/co-expression leads to exhaustion.	Galectin-9, CEACAM1	(12, 14, 24, 52)
CD8^+^ T Cells	Regulates cytotoxicity, cytokine production, and exhaustion.	COPD: Subset-specific regulation (Tc1 vs. Tc17); co-expression with PD-1 marks exhaustion. Lung Cancer: Tim-3^+^PD-1^+^ marks terminally exhausted TILs. TB: Associated with exhausted phenotype in MDR-TB.	Galectin-9, CEACAM1	(14, 29, 48, 62)
Regulatory T Cells (Tregs)	Often associated with enhanced suppressive capacity.	ARDS: Tim-3^+^Tregs alleviate injury via STAT3-IL-10 axis. Pulmonary Fibrosis: Tim-3^+^Tregs exert anti-fibrotic effects. COPD: Loss of Gal-9 on Tregs impairs regulation of Th17.	Galectin-9	(15, 30, 36)
Macrophages	Polarization regulation; phagocytosis.	COPD: Promotes M2 polarization (potential vitamin D link). ARDS: Tim-3/Gal-9 axis time-dependently regulates M1/M2. Pulmonary Fibrosis: Dual role: pro-fibrotic (secretory) vs. pro-resolution (phagocytic).	Galectin-9, PtdSer	(31, 38, 44, 45)
Dendritic Cells (DCs)	Modulates antigen presentation and immune priming.	Lung Cancer: Inhibits cGAS-STING pathway, impairing function. TB: M. tb exploits Tim-3^+^ DCs to generate immunosuppressive MDSCs.	HMGB1, Galectin-9	(54, 63)
Mast Cells	Role in allergic inflammation; precise mechanism unclear.	Asthma/CRSwNP: Tim-3^+^ mast cells in epithelium correlate with disease severity.	Under investigation	(20)
Innate Lymphoid Cells (e.g., MAITs)	May mark activated, tissue-resident effector subsets.	TB: Tim-3^+^ tissue-resident MAITs in pleura have enhanced effector function.	Cytokine-regulated (IL-12, IL-18)	(53)

The second is cell type specificity: in the same disease, the function of Tim-3 on different cells varies significantly. For example, in pulmonary fibrosis, Tim-3 on alveolar macrophages can promote fibrosis or mediate apoptotic cell clearance, while Tim-3 on Tregs clearly exerts an anti-fibrotic effect; in COPD, Tim-3 on CD8^+^T cells shows opposite regulatory effects on Tc1 and Tc17 subsets, with no obvious inhibition on the pro-inflammatory function of Tc1 but inhibition on Tc17 cells. The third is stage specificity: blocking Tim-3 in the precancerous lesion stage of lung cancer has a significant effect, while combination therapy is needed in the late stage; Tim-3 is highly expressed in the stable phase of COPD and lowly expressed in the acute exacerbation phase, which is consistent with the stage-specific needs of inflammation regulation.

### 8.2 Challenges and outlook

Current studies on Tim-3 in respiratory diseases still face many challenges. At the mechanism research level, it is necessary to further clarify the precise signaling pathways of Tim-3 in different cell subsets and their intercellular crosstalk mechanisms, such as the regulatory details of the NFIL3/Tim-3 axis in COPD T cells and the molecular switch for Tim-3 function switching in alveolar macrophages. The clarification of these mechanisms is the basis for precise targeting. At the biomarker development level, it is necessary to explore the value of plasma soluble Tim-3 (sTim-3) or cellular Tim-3 expression levels in disease diagnosis, typing, and prognosis evaluation, such as the differentiation between MDR-TB and NR-TB and the prediction of ICI treatment efficacy in lung cancer. At the same time, standardized detection methods need to be established to reduce errors caused by detection differences (**Table 3**).

**Table 3:** Key mechanistic axes and targeted strategies involving tim-3.

Core axis / pathway	Biological context	Net effect	Targeted strategies (preclinical/clinical)	Ref.
Tim-3 / Galectin-9	Asthma: On CD4^+^ T cells. COPD: On Tregs & effector cells. ARDS: Time-dependent on macrophages. TB: On T cells and macrophages.	Predominantly immunosuppressive. Inhibits Th1, promotes Treg function, regulates macrophage polarization.	Blockade: In TB to reverse exhaustion. Modulation: In ARDS/COPD to regulate inflammation (challenging).	(13, 19, 30, 37, 38, 51)
Tim-3 / CEACAM1	Lung Cancer: Epithelial-myeloid crosstalk in precancerous lesions. ARDS: On neutrophils in sepsis.	Promotes immunosuppressive niche formation (cancer); drives NETosis (sepsis).	Blockade: For precancerous interception (cancer). Inhibition: e.g., Xuebijing to reduce NETs (sepsis-ALI).	(16, 39)
Tim-3^+^ Treg / STAT3-IL-10	ARDS: Resolution phase. Pulmonary Fibrosis.	Anti-inflammatory & anti-fibrotic. Promotes M2 macrophage polarization.	Adoptive Cell Therapy: Transfer of Tim-3^+^ Tregs. Pharmacologic enhancement: Of this axis.	(15)
NFIL3 / Tim-3	COPD: In CD4^+^ T cells.	Upstream transcriptional regulation of Tim-3, controlling Th1 inflammation.	Potential target upstream of Tim-3 (exploratory).	(32)
Tim-3 & PD-1 Co-expression	Lung Cancer, TB, COPD: On exhausted CD8^+^ and CD4^+^ T cells.	Marker of terminal T cell exhaustion; driver of ICI resistance.	Combination Blockade: Anti-Tim-3 + anti-PD-1/L1 (clinical trials in NSCLC, potential in TB).	(48, 62, 67)
Hypoxia / HIF-1α / Gal-9 / Tim-3	Lung Cancer with OSA.	Upregulates checkpoint ligands, exacerbating immunosuppression.	Managing hypoxia; potentially targeting the axis.	(65)

At the targeted therapy development level, first, it is necessary to optimize the efficacy and safety of anti-Tim-3 antibody drugs to solve the problem of limited monotherapy efficacy; second, active exploration of combination therapy strategies with other immune checkpoint inhibitors (such as PD-(L)1 inhibitors), chemotherapy, radiotherapy, etc., such as the combination of anti-Tim-3 and anti-PD-1 in lung cancer to address compensatory resistance; most importantly, the “double-edged sword” effect of Tim-3 in different diseases needs to be fully considered to avoid risks caused by excessive blocking or activation. For example, in pulmonary infections, it is necessary to distinguish between the T cell exhaustion stage and the acute infection stage; in pulmonary fibrosis, it is necessary to distinguish between different functions of macrophages. Personalized treatment can be achieved through precise stratification. In the future, with the in-depth development of mechanism research and the optimization of targeted strategies, Tim-3 is expected to become an important molecular target for the diagnosis and treatment of respiratory diseases, providing new ideas and methods for improving patient prognosis.

## Data Availability

The original contributions presented in the study are included in the article/supplementary material. Further inquiries can be directed to the corresponding authors.

## References

[1] Kikushige Y . TIM-3 in normal and Malignant hematopoiesis: Structure, function, and signaling pathways. Cancer Sci. (2021) 112:3419–26. doi: 10.1111/cas.15042, PMID: 34159709 PMC8409405

[2] Cheng B Lv J Xiao Y Song C Chen J Shao C . Small molecule inhibitors targeting PD-L1, CTLA4, VISTA, TIM-3, and LAG3 for cancer immunotherapy (2020-2024). Eur J Med Chem. (2025) 283:117141. doi: 10.1016/j.ejmech.2024.117141, PMID: 39653621

[3] Guo X Yu S Tao J Wang Y Shao Z Fu R . Advances in the study of TIM3 in myelodysplastic syndrome. Front Immunol. (2025) 16:1647401. doi: 10.3389/fimmu.2025.1647401, PMID: 40904469 PMC12401906

[4] Sauer N Janicka N Szlasa W Skinderowicz B Kołodzińska K Dwernicka W . TIM-3 as a promising target for cancer immunotherapy in a wide range of tumors. Cancer Immunol Immunother. (2023) 72:3405–25. doi: 10.1007/s00262-023-03516-1, PMID: 37567938 PMC10576709

[5] Zhao L Cheng S Fan L Zhang B Xu S . TIM-3: An update on immunotherapy. Int Immunopharmacol. (2021) 99:107933. doi: 10.1016/j.intimp.2021.107933, PMID: 34224993

[6] Widyagarini A Nishii N Kawano Y Zhang C Azuma M . VSIG4/CRIg directly regulates early CD8(+) T cell activation through its counter-receptor in a narrow window. Biochem Biophys Res Commun. (2022) 614:100–6. doi: 10.1016/j.bbrc.2022.04.120, PMID: 35576680

[7] Pang N Alimu X Chen R Muhashi M Ma J Chen G . Activated Galectin-9/Tim3 promotes Treg and suppresses Th1 effector function in chronic lymphocytic leukemia. FASEB J. (2021) 35:e21556. doi: 10.1096/fj.202100013R, PMID: 34137463

[8] Galluzzi L Galassi C Wiest DL . TIM-3 and γδ T cells: new players in breast cancer dissemination. EMBO J. (2025) 44:5236–8. doi: 10.1038/s44318-025-00550-w, PMID: 40859036 PMC12489074

[9] Detsika MG Palamaris K Dimopoulou I Kotanidou A Orfanos SE . The complement cascade in lung injury and disease. Respir Res. (2024) 25:20. doi: 10.1186/s12931-023-02657-2, PMID: 38178176 PMC10768165

[10] de Fays C Geudens V Gyselinck I Kerckhof P Vermaut A Goos T . Mucosal immune alterations at the early onset of tissue destruction in chronic obstructive pulmonary disease. Front Immunol. (2023) 14:1275845. doi: 10.3389/fimmu.2023.1275845, PMID: 37915582 PMC10616299

[11] Virk H Arthur G Bradding P . Mast cells and their activation in lung disease. Transl Res. (2016) 174:60–76. doi: 10.1016/j.trsl.2016.01.005, PMID: 26845625

[12] Tang F Wang F An L Wang X . Upregulation of Tim-3 on CD4(+) T cells is associated with Th1/Th2 imbalance in patients with allergic asthma. Int J Clin Exp Med. (2015) 8:3809–16. PMC4443112 26064278

[13] Seki M Oomizu S Sakata KM Sakata A Arikawa T Watanabe K . Galectin-9 suppresses the generation of Th17, promotes the induction of regulatory T cells, and regulates experimental autoimmune arthritis. Clin Immunol. (2008) 127:78–88. doi: 10.1016/j.clim.2008.01.006, PMID: 18282810

[14] Ke J Huang S He Z Lei S Lin S Duan M . Integrated bioinformatic analysis and experimental validation for exploring the key immune checkpoint of COPD. Gene. (2024) 927:148711. doi: 10.1016/j.gene.2024.148711, PMID: 38906393

[15] Liu X Jiang S Zhang Q Xu S Bao X Cao W . Tim-3 regulates tregs’ Ability to resolve the inflammation and proliferation of acute lung injury by modulating macrophages polarization. Shock. (2018) 50:455–64. doi: 10.1097/SHK.0000000000001070, PMID: 29194342

[16] Zhu B Chen P Aminu M Li JR Fujimoto J Tian Y . Spatial and multiomics analysis of human and mouse lung adenocarcinoma precursors reveals TIM-3 as a putative target for precancer interception. Cancer Cell. (2025) 43:1125–1140.e10. doi: 10.1016/j.ccell.2025.04.003, PMID: 40345189 PMC12151776

[17] Ghrairi N Elhechmi YZ . Physiopathology of allergic asthma: A comprehensive review. Scand J Immunol. (2025) 101:e70032. doi: 10.1111/sji.70032, PMID: 40401813

[18] Lambrecht BN Ahmed E Hammad H . The immunology of asthma. Nat Immunol. (2025) 26:1233–45. doi: 10.1038/s41590-025-02212-9, PMID: 40730897

[19] Mosayebian A Koohini Z Hossein-Nataj H Abediankenari S Abedi S Asgarian-Omran H . Elevated expression of tim-3 and PD-1 immune checkpoint receptors on T-CD4+ Lymphocytes of patients with asthma. Iran J Allergy Asthma Immunol. (2018) 17:517–25. 30644695

[20] Hamada K Yoshimura K Oshinomi K Hirasawa Y Ariizumi H Ohkuma R . A case of bronchial asthma as an immune-related adverse event of pembrolizumab treatment for bladder cancer: A case report. Med (Baltimore). (2022) 101:e28339. doi: 10.1097/MD.0000000000028339, PMID: 35029177 PMC8757941

[21] Sadri M Ganjalikhani-Hakemi M Akbari P Salehi R Rastaghi S Ghasemi R . Association between +4259 T>G and -574 G>T polymorphisms of TIM-3 with asthma in an Iranian population. Iran J Allergy Asthma Immunol. (2017) 16:321–8. 28865412

[22] Wei W Huang J Ma Y Hao C Zhang S . Association between gene polymorphisms of T cell immunoglobulin domain and mucin domain-3 and risk of asthma: A systematic review and meta-analysis. Iran J Allergy Asthma Immunol. (2021) 20:1–10. doi: 10.18502/ijaai.v20i1.5407, PMID: 33639624

[23] Maolahong S Chen JH Cai XT Cong L Chen Y Lian ZC . Genetic polymorphisms of TIM-3 and its association with asthma in familial cluster asthma. Postepy Dermatol Alergol. (2025) 42:267–75. doi: 10.5114/ada.2025.152152, PMID: 40672727 PMC12262037

[24] Chen X Song CH Liu ZQ Feng BS Zheng PY Li P . Intestinal epithelial cells express galectin-9 in patients with food allergy that plays a critical role in sustaining allergic status in mouse intestine. Allergy. (2011) 66:1038–46. doi: 10.1111/j.1398-9995.2011.02585.x, PMID: 21426359

[25] Boehne C Behrendt AK Meyer-Bahlburg A Boettcher M Drube S Kamradt T . Tim-3 is dispensable for allergic inflammation and respiratory tolerance in experimental asthma. PloS One. (2021) 16:e0249605. doi: 10.1371/journal.pone.0249605, PMID: 33822811 PMC8023500

[26] Xu J Zeng Q Li S Su Q Fan H . Inflammation mechanism and research progress of COPD. Front Immunol. (2024) 15:1404615. doi: 10.3389/fimmu.2024.1404615, PMID: 39185405 PMC11341368

[27] Qi Y Yan Y Tang D Han J Zhu X Cui M . Inflammatory and immune mechanisms in COPD: current status and therapeutic prospects. J Inflammation Res. (2024) 17:6603–18. doi: 10.2147/JIR.S478568, PMID: 39318994 PMC11421452

[28] Gémes N Balog J Neuperger P Schlegl E Barta I Fillinger J . Single-cell immunophenotyping revealed the association of CD4+ central and CD4+ effector memory T cells linking exacerbating chronic obstructive pulmonary disease and NSCLC. Front Immunol. (2023) 14:1297577. doi: 10.3389/fimmu.2023.1297577, PMID: 38187374 PMC10770259

[29] Song D Yan F Fu H Li L Hao J Zhu Z . A cellular census of human peripheral immune cells identifies novel cell states in lung diseases. Clin Transl Med. (2021) 11:e579. doi: 10.1002/ctm2.579, PMID: 34841705 PMC8611783

[30] Qiu S Zhou G Ke J Zhou J Zhang H Jin Z . Impairment of Gal-9 and Tim-3 crosstalk between Tregs and Th17 cells drives tobacco smoke-induced airway inflammation. Immunology. (2024) 173:152–71. doi: 10.1111/imm.13820, PMID: 38829009

[31] Liang S Cai J Li Y Yang R . 1,25−Dihydroxy−Vitamin D3 induces macrophage polarization to M2 by upregulating T−cell Ig−mucin−3 expression. Mol Med Rep. (2019) 19:3707–13. doi: 10.3892/mmr.2019.10047, PMID: 30896850 PMC6472136

[32] Ke J Huang S He Z Lei S Lin S Li Y . NFIL3/Tim3 axis regulates effector Th1 inflammation in COPD mice. Front Immunol. (2024) 15:1482213. doi: 10.3389/fimmu.2024.1482213, PMID: 39555065 PMC11563780

[33] Li A Zhang K Wang H Li J Yao Y Tu YS . Integrative transcriptomic analysis reveals cross-species conserved core genes and pathways in alveolar macrophages during ALI/ARDS. BMC Pulm Med. (2025) 25:447. doi: 10.1186/s12890-025-03928-y, PMID: 41039310 PMC12492643

[34] Zhang M Shang L Zhou F Cai Y Wang S Li J . Targeting PANoptosis: a promising therapeutic strategy for ALI/ARDS. Apoptosis. (2025) 30:2547–87. doi: 10.1007/s10495-025-02168-z, PMID: 40906270

[35] Ziaka M Exadaktylos A . Acute respiratory distress syndrome: pathophysiological insights, subphenotypes, and clinical implications-A comprehensive review. J Clin Med. (2025) 14:5184. doi: 10.3390/jcm14155184, PMID: 40806804 PMC12347897

[36] Song H Zhou Y Li G Bai J . Regulatory T cells contribute to the recovery of acute lung injury by upregulating Tim-3. Inflammation. (2015) 38:1267–72. doi: 10.1007/s10753-014-0096-7, PMID: 25526715

[37] Liu J Xiao S Huang S Pei F Lu F . Upregulated Tim-3/galectin-9 expressions in acute lung injury in a murine malarial model. Parasitol Res. (2016) 115:587–95. doi: 10.1007/s00436-015-4775-6, PMID: 26494364 PMC7101834

[38] Zhang W Zhang Y He Y Wang X Fang Q . Lipopolysaccharide mediates time-dependent macrophage M1/M2 polarization through the Tim-3/Galectin-9 signalling pathway. Exp Cell Res. (2019) 376:124–32. doi: 10.1016/j.yexcr.2019.02.007, PMID: 30763585

[39] Zhang S Jiang M Wu C Wang H He F . Modulation of the CEACAM1/TIM3 pathway by traditional Chinese medicine Xuebijing alleviates sepsis-induced acute lung injury through inhibition of NETs formation. J Ethnopharmacol. (2025) 353:120280. doi: 10.1016/j.jep.2025.120280, PMID: 40651727

[40] Song Q Lin L Chen L Cheng L Zhong W . Co-administration of N-acetylcysteine and dexmedetomidine plays a synergistic effect on protection of LPS-induced acute lung injury via correcting Th1/Th2/Th17 cytokines imbalance. Clin Exp Pharmacol Physiol. (2020) 47:294–301. doi: 10.1111/1440-1681.13196, PMID: 31631367

[41] Liu J Huang S Su XZ Song J Lu F . Blockage of galectin-receptor interactions by α-lactose exacerbates plasmodium berghei-induced pulmonary immunopathology. Sci Rep. (2016) 6:32024. doi: 10.1038/srep32024, PMID: 27554340 PMC4995515

[42] Pan T Feng Y Li Y Yang Y Zhou J Song Y . Exacerbation of pulmonary fibrosis following acute lung injury via activin-A production by recruited alveolar macrophages. J Thorac Dis. (2024) 16:7709–28. doi: 10.21037/jtd-24-680, PMID: 39678869 PMC11635239

[43] Koudstaal T Funke-Chambour M Kreuter M Molyneaux PL Wijsenbeek MS . Pulmonary fibrosis: from pathogenesis to clinical decision-making. Trends Mol Med. (2023) 29:1076–87. doi: 10.1016/j.molmed.2023.08.010, PMID: 37716906

[44] Wang Y Kuai Q Gao F Wang Y He M Zhou H . Overexpression of TIM-3 in macrophages aggravates pathogenesis of pulmonary fibrosis in mice. Am J Respir Cell Mol Biol. (2019) 61:727–36. doi: 10.1165/rcmb.2019-0070OC, PMID: 31162951

[45] Isshiki T Akiba H Nakayama M Harada N Okumura K Homma S . Cutting edge: anti-TIM-3 treatment exacerbates pulmonary inflammation and fibrosis in mice. J Immunol. (2017) 199:3733–7. doi: 10.4049/jimmunol.1700059, PMID: 29061768

[46] Scriba TJ Maseeme M Young C Taylor L Leslie AJ . Immunopathology in human tuberculosis. Sci Immunol. (2024) 9:eado5951. doi: 10.1126/sciimmunol.ado5951, PMID: 39671470

[47] Korkmaz FT Traber KE . Innate immune responses in pneumonia. Pneumonia (Nathan). (2023) 15:4. doi: 10.1186/s41479-023-00106-8, PMID: 36829255 PMC9957695

[48] Yang X Yao L Gui XW Lai Y Ji P Wang Y . Immune profiles of immune checkpoint molecules on peripheral T cells in multidrug-resistant tuberculosis. Int J Infect Dis. (2025) 161:108085. doi: 10.1016/j.ijid.2025.108085, PMID: 41038528

[49] Liu CW Wu LS Lin CJ Wu HC Liu KC Lee SW . Association of tuberculosis risk with genetic polymorphisms of the immune checkpoint genes PDCD1, CTLA-4, and TIM3. PloS One. (2024) 19:e0303431. doi: 10.1371/journal.pone.0303431, PMID: 38723011 PMC11081348

[50] Sharma K Sharma A Arora SK . Mycobacterium tuberculosis modulates the expansion of terminally exhausted CD4(+) and CD8(+) T-cells in individuals with HIV-TB co-infection. Pathog. (2025) 14:802. doi: 10.3390/pathogens14080802, PMID: 40872312 PMC12389345

[51] Kang J Wei ZF Li MX Wang JH . Modulatory effect of Tim-3/Galectin-9 axis on T-cell-mediated immunity in pulmonary tuberculosis. J Biosci. (2020) 45:60. doi: 10.1007/s12038-020-0023-z, PMID: 32345786

[52] Jean Bosco M Wei M Hou H Yu J Lin Q Luo Y . The exhausted CD4(+)CXCR5(+) T cells involve the pathogenesis of human tuberculosis disease. Int J Infect Dis. (2018) 74:1–9. doi: 10.1016/j.ijid.2018.06.011, PMID: 29936320

[53] Jiang J Cao Z Xiao L Li B Yu S Yang B . Tim-3 expression is induced by mycobacterial antigens and identifies tissue-resident subsets of MAIT cells from patients with tuberculosis. Microbes Infect. (2023) 25:105021. doi: 10.1016/j.micinf.2022.105021, PMID: 35811063

[54] Singh S Maurya SK Aqdas M Bashir H Arora A Bhalla V . Mycobacterium tuberculosis exploits MPT64 to generate myeloid-derived suppressor cells to evade the immune system. Cell Mol Life Sci. (2022) 79:567. doi: 10.1007/s00018-022-04596-5, PMID: 36283989 PMC11803053

[55] Chávez-Galán L Ramon-Luing L Carranza C Garcia I Sada-Ovalle I . Lipoarabinomannan decreases galectin-9 expression and tumor necrosis factor pathway in macrophages favoring mycobacterium tuberculosis intracellular growth. Front Immunol. (2017) 8:1659. doi: 10.3389/fimmu.2017.01659, PMID: 29230224 PMC5711832

[56] Koh J Kim S Kim JY Yim JJ Kwak N . Immunologic features of nontuberculous mycobacterial pulmonary disease based on spatially resolved whole transcriptomics. BMC Pulm Med. (2024) 24:392. doi: 10.1186/s12890-024-03207-2, PMID: 39138424 PMC11323347

[57] Wu G Sun X . Thymosin α1 combined with 2HRZE/4HR regimen as a potential treatment of pulmonary tuberculosis: an analysis of immune function, pulmonary function and inflammatory response. Br J Hosp Med (Lond). (2025) 86:1–14. doi: 10.12968/hmed.2025.0235, PMID: 40994373

[58] Antas PRZ da Silva ASM Albuquerque LHP Almeida MR Pereira E Castello-Branco LRR . The BCG moreau vaccine upregulates *in vitro* the expression of TLR4, B7-1, dectin-1 and EP2 on human monocytes. Vaccines (Basel). (2022) 11:86. doi: 10.3390/vaccines11010086, PMID: 36679931 PMC9861981

[59] Wang Z Guo H Song Y Wang A Yan Y Ma L . Lung cancer tumor immune microenvironment: analyzing immune escape mechanisms and exploring emerging therapeutic targets. Front Immunol. (2025) 16:1597686. doi: 10.3389/fimmu.2025.1597686, PMID: 41080606 PMC12507600

[60] Liu L Yang L Li H Shang T Liu L . The tumor microenvironment in lung cancer: Heterogeneity, therapeutic resistance and emerging treatment strategies (Review). Int J Oncol. (2026) 68:11. doi: 10.3892/ijo.2025.5824, PMID: 41312736 PMC12674202

[61] Liu Y Qin D Fu J . T lymphocyte heterogeneity in NSCLC: implications for biomarker development and therapeutic innovation. Front Immunol. (2025) 16:1604310. doi: 10.3389/fimmu.2025.1604310, PMID: 40510347 PMC12159075

[62] Kabut J Gorzelak-Magiera A Gisterek-Grocholska I New Therapeutic Targets TIGIT . LAG-3 and TIM-3 in the treatment of advanced, non-small-cell lung cancer. Int J Mol Sci. (2025) 26:4096. doi: 10.3390/ijms26094096, PMID: 40362333 PMC12072094

[63] Guégan JP Peyraud F Dadone-Montaudie B Teyssonneau D Palmieri LJ Clot E . Analysis of PD1, LAG3, TIGIT, and TIM3 expression in human lung adenocarcinoma reveals a 25-gene signature predicting immunotherapy response. Cell Rep Med. (2024) 5:101831. doi: 10.1016/j.xcrm.2024.101831, PMID: 39591972 PMC11722093

[64] Ying X Lou Y Wu Y Hu W . Integrated computational and experimental identification of N-[(1H-1,2,4-triazol-3-yl)phenyl]-1-(1H-pyrazolo[3,4-b]pyridin-3-yl)methanamide as a potent and selective TIM-3 inhibitor for NSCLC immunotherapy. Front Chem. (2025) 13:1622511. doi: 10.3389/fchem.2025.1622511, PMID: 40552075 PMC12183220

[65] Díaz-García E Alfaro E Pérez-Moreno P López-Fernández C García-Sánchez A Martínez-García M . Immune checkpoint biomarkers galectin-9 and TIM-3 predict melanoma and lung cancer mortality in obstructive sleep apnoea. Arch Bronconeumol. (2025) 61:735–48. doi: 10.1016/j.arbres.2025.03.018, PMID: 40287376

[66] Kang L Cao J Guo W Cui X Wei Y Zhang J . Tumor necrosis factor-α-dependent inflammation upregulates high mobility group box 1 to induce tumor promotion and anti-programmed cell death protein-1 immunotherapy resistance in lung adenocarcinoma. Lab Invest. (2025) 105:102164. doi: 10.1016/j.labinv.2024.102164, PMID: 39461427

[67] Davar D Eroglu Z Milhem M Becerra C Gutierrez M Ribas A . Combined targeting of PD-1 and TIM-3 in patients with locally advanced or metastatic non-small cell lung cancer: AMBER part 2B. Clin Cancer Res. (2025) 31:3443–51. doi: 10.1158/1078-0432.CCR-25-0806, PMID: 40552922 PMC12351275

